# Associations of blood biomarkers with glomerular filtration rate in patients with TIA and stroke: population-based study

**DOI:** 10.1136/svn-2020-000422

**Published:** 2020-09-03

**Authors:** Dearbhla M. Kelly, Linxin Li, Annette I Burgess, Deborah L Poole, Julia M Duerden, Peter M. Rothwell

**Affiliations:** Wolfson Centre for the Prevention of Stroke and Dementia, Nuffield Department of Clinical Neurosciences, Oxford University, Oxford, Oxfordshire, UK

**Keywords:** stroke, blood pressure, brain, atherosclerosis

## Abstract

**Background and purpose:**

Non-traditional risk factors such as chronic inflammation, oxidative stress and thrombogenic factors are believed to contribute to the excess stroke risk in chronic kidney disease (CKD) by triggering vascular injury and endothelial dysfunction. We aimed to determine how well a panel of biomarkers representative of these factors would correlate with estimated glomerular filtration rate (eGFR) in patients with recent transient ischaemic attack (TIA) or stroke. We also investigated whether eGFR would confound previously reported associations between biomarkers and mortality.

**Methods:**

We studied a panel of 16 blood biomarkers related to inflammation, thrombosis, atherogenesis and cardiac or neuronal cell damage in TIA or ischaemic stroke in a population-based study (Oxford Vascular Study). Biomarker levels were log-transformed and correlated with eGFR, adjusted for age. Cox proportional hazard models were used for survival analysis.

**Results:**

Among 1297 patients with TIA or stroke, 52.7% (n=684) of patients had CKD (eGFR <60 mL/min/1.73 m^2^). There was a moderate correlation between log-eGFR and the log-transformed soluble tumour necrosis factor receptor-1 (R^2^=0.21), attenuating with adjustment for age (R^2^=0.12). There were moderate-to-strong correlations with markers of cardiac injury, N-terminal pro-brain natriuretic peptide and heart-type fatty acid binding protein (hFABP, R^2^=0.14 and 0.34, respectively). The strongest correlation after adjustment for age was between hFABP and eGFR (R^2^=0.20). Adjusting for eGFR did not impact any biomarker associations with mortality.

**Conclusions:**

Correlations between biomarkers related to inflammation and thrombosis with renal dysfunction in the setting of cerebrovascular events were generally modest after adjustment for age, suggesting that putative risk factors such as chronic inflammation or coagulopathy are unlikely to be important stroke mechanisms in patients with CKD.

## Introduction

Chronic kidney disease (CKD) affects as many as 10%–15% of the population worldwide.^1^ It is a rapidly growing global health burden, mainly because it is an established risk factor for cardiovascular disease and stroke.^2^ Meta-analyses of cohort studies and trials indicate that reduced glomerular filtration rate (GFR) increases the risk of stroke by about 40%^3^ and that proteinuria increases the risk up to 70%^4^ even after adjusting for traditional cardiovascular risk factors.

Traditional cardiovascular risk factors, including hypertension,^5^ diabetes mellitus^6^ and atrial fibrillation,^7^ are all highly prevalent in the CKD population, likely confounding much of the association between CKD and stroke. However, unconventional risk factors directly resulting from renal disease, such as chronic inflammation, oxidative stress and thrombogenic factors, are also proposed to contribute to the excess cerebrovascular risk observed in patients with CKD by triggering vascular injury and endothelial dysfunction.^8 9^

Use of blood biomarkers related to these potential disease pathways of inflammation,^10^ coagulation,^11^ atherogenesis,^12^ cardiac or neuronal injury^13 14^ has been studied in the general population to aid stroke diagnosis, determine subtype or mechanism and to predict outcome or response to therapy.^13 15–17^ In patients with CKD, inflammatory biomarkers (including interleukin 6 (IL-6), tumour necrosis factor α (TNF-α), high-sensitivity C-reactive protein (CRP), fibrinogen and serum albumin) have been shown to be independently associated with incident atherosclerotic events and death, suggesting a possible causative role for inflammation in cardiovascular events in CKD.^18^ However, previous studies have not had a large population-based cohort design, have not focused specifically on stroke and have included only a narrow range of biomarkers.

Similar biomarker associations with mortality have been shown in patients with transient ischaemic attack (TIA) and stroke in the general population; however, they have not previously been adjusted for renal function, and thus CKD or its associated renal vascular risk factors may be a confounder in this relationship.^19^

We therefore aimed to investigate in a large prospective population-based study of patients with TIA or stroke, the correlations of a broad panel of biomarkers related to inflammation, thrombosis and cardiac or neuronal function or injury with renal function. We hypothesised that if non-traditional risk factors such as CKD-related coagulopathy or inflammation are aetiologically important in stroke pathogenesis, then representative biomarkers should independently correlate with GFR in this setting. Furthermore, we also aimed to determine if previously reported biomarker-mortality associations^19^ are confounded by renal dysfunction.

## Materials and methods

### Patients

OxVASC is an ongoing population-based study of the incidence and outcome of cerebrovascular, cardiovascular and peripheral vascular events since 2002. The study population comprises all 92 728 individuals, irrespective of age, registered with about 100 family physicians in nine primary care practices in Oxfordshire. The methods have been approved by the Oxfordshire Research Ethics Committee. Multiple methods of ascertainment are used to ascertain patients with TIA or stroke, as detailed elsewhere.^20^ Briefly, multiple overlapping methods of hot and cold pursuit are used to achieve near-complete ascertainment of all individuals with TIA or stroke. These include (1) a daily, rapid access TIA clinic to which participating general practitioners and the local accident and emergency department refer all individuals with suspected, but not hospitalised, TIA or stroke; (2) daily searches of admissions to the medical, stroke, neurology and other relevant wards; (3) daily searches of the local accident and emergency department attendance register; (4) daily searches of in-hospital death records via the Bereavement Office; (5) monthly searches of all death certificates and coroner’s reports for out-of-hospital deaths; (6) monthly searches of general practitioner diagnostic coding and hospital discharge codes; and (7) monthly searches of all vascular imaging referrals.

All patients provided written informed consent or assent was obtained from relatives, and they were seen by study physicians as soon as possible after their initial presentation. A standardised questionnaire was used to record a comprehensive medical history. The severity of events was assessed using the National Institute of Health Stroke Scale (NIHSS)^21^ and clinical features. The study senior neurologist (PR) reviewed all cases and classified them as stroke or other condition using standard definitions.^22^ Events were classified as minor stroke if there was a focal neurological deficit lasting >24 hours and an NIHSS score ≤3 at time of assessment by a study physician. All patients were followed up by a research nurse or physician at 1, 3, 6, 12, 24, 60 and 120 months after the index event. Recurrent ischaemic events, bleeding events, and disability (modified Rankin Scale) were recorded at each follow-up visit.

Non-fasting blood samples were taken as soon as possible after the event, usually within a couple of days. These included serum, 3.2% buffered tri-sodium citrate plasma and lithium heparin plasma (Vacutainer tubes; Becton Dickinson, UK). Samples were centrifuged at 3000 g for 10 min, and aliquots of serum and plasma were stored at −80°C before analysis when they were thawed for use at 37°C. All times from sampling to freezing were documented, typically within 4 hours of taking.

CKD was defined as eGFR <60 mL/min/1.73 m^2^ for 3 or more months as per 2012 Kidney Disease: Improving Global Outcomes guidelines.^23^ eGFR was estimated using the Chronic Kidney Disease Epidemiology Collaboration Equation. However, we also performed a sensitivity analysis of eGFR-biomarker correlations using the Full Age Spectrum equation (eGFR-FAS) to estimate GFR as this has been proposed to provide better continuity of prediction at extremes of ages.^24^ We reviewed all available premorbid creatinine values for each patient to ensure that they fulfilled the duration criteria for a definition of a CKD.

Deaths were classified blind to biomarker results after review of clinical records and death certificates available from the study practices. Practice-specific listings of all *International Classification of Diseases, Tenth Revision* death codes were also obtained from central registers.

### Assay methods

Our assay methods which have been previously described, included a mixture of chip systems and separate ELISA assays.^17 19^ Briefly, a Biochip multiple immunoassay system (Randox Laboratories Ltd, Co, Antrim, Northern Ireland) using lithium-heparin simultaneously measured biomarkers via two panels: Cerebral Array I for brain-derived neurotrophic factor (BDNF), IL-6 and heart-type fatty acid-binding protein (hFABP), and Cerebral Array II for CRP, neurone-specific enolase, neutrophil gelatinase-associated lipocalin (NGAL), soluble tumour necrosis factor receptor-1 (sTNF-R1) and thrombomodulin (TM). All assays underwent reproducibility studies using an internal control of pooled normal lithium-heparin plasma in addition to control materials supplied by the manufacturers.

Citrate plasma was used for measuring the thrombotic markers fibrinogen, D-dimer and von Willebrand Factor (vWF) antigen by immunoturbidometric assays (Sta-Liatest D-Dimer, DiagnosticaStago, Asniers, France) using a diagnostic automated coagulation analyser STA (Diagnostica Stago). These assays have well-defined internal quality control and external control through participation in national quality assurance schemes. Commercial ELISA kits were used to assay human P-selectin (R&D Systems, UK), Protein Z (PZ, DiagnosticaStago, France) and NT-proBNP (Biomedica, Austria). Serum samples were used for anti-phosphorylcholine (anti-PC) antibodies (CVDefine; Athera Biotechnologies AB, Stockholm, Sweden), adhering to manufacturers recommendations. Percentage of intracoefficients and intercoefficients of variation of each assay is shown in online supplemental table 1. All assays were performed blind to study status. During the study period, serum creatinine was measured using a compensated Jaffe method which was IDMS traceable.

10.1136/svn-2020-000422.supp1 Supplementary data

### Statistical analysis

Continuous data were reported as mean (SD) or median (IQR) as appropriate, categorical data were reported as n (%). Independent-samples t-tests and χ^2^ tests were used to test the significance of differences between two groups for continuous and categorical variables, respectively. One-way analysis of variance (ANOVA) was used to compare differences between more than two groups. A Mann-Whitney U test and a Kruskall-Wallis test were used as non-parametric alternatives for t-tests and one-way ANOVA, respectively. Age-adjusted p values for difference were calculated using logistic and linear regression, where appropriate. Correlations of biomarkers and GFR were calculated by Spearman rank correlations. To further evaluate potentially linear relationships, biomarkers and GFR were then both log-transformed and correlated using Pearson correlations. Partial correlations were used to adjust for age. Subgroup analysis was performed according to age category, sex and hypertension status. Correlation coefficients were compared using Fisher’s Z transformations and the χ^2^ test of heterogeneity. For survival analysis, Cox proportional hazard models were used. Associations of biomarkers with all-cause death were calculated unadjusted, adjusted for age and sex (model 1); adjusted for variables included in model 1 plus eGFR (model 2); and adjusted for variables included in model 2 plus hypertension, diabetes, atrial fibrillation, prior myocardial infarction or ischaemic stroke, peripheral artery disease, hyperlipidaemia, smoking, prior therapy with antiplatelet agents, antihypertensive agents and statins (model 3). Results were considered significant at p<0.05. All statistical analyses were performed using SPSS V.25.0.

## Results

### Baseline clinical and demographic characteristics

A total of 1297 consecutive eligible patients with TIA (n=427), minor stroke (n=549) or major stroke (n=321) were recruited from 2002 to 2011, and followed up until 2013. Table 1 shows the baseline characteristics at the time of the event for all patients and according to CKD status. The median age (IQR) was 75.2 (65.2–83.2) years, 47.8% (n=620) were men, and hypertension was the most prevalent risk factor being found in 59.1% (n=766). The median (IQR) days from event to sampling were 5 (3–12) days. A maximum of 1210 samples were analysed by the Randox panels with numbers limited in certain cases by inadequate sample volumes and assay failure. Thrombotic markers were measured in a maximum of 1213 patients after exclusion of cases with inadequate sample volumes and anticoagulant therapy. The median eGFR was 58.7 (46.7–70.6) mL/min/1.73 m^2^. A total of 684 (52.7%) patients had CKD, 637 (93.1%) of whom had stage 3 (eGFR=30–59 mL/min/1.73 m^2^), 64 (6.4%) had stage 4 (eGFR=15–29 mL/min/1.73 m^2^) and 3 (0.4%) had stage 5 (eGFR <15 mL/min/1.73 m^2^).

**Table 1:** Baseline characteristics of all patients with TIA and stroke, and stratified according to the presence of CKD

Characteristics*	All patients n=1297	No CKD n=607	CKD n=684	P value†	Age-adjusted p value
Age years, median (IQR)	75.2 (65.2–83.2)	67.3 (58.5–77.5)	80.3 (72.7–85.2)	<0.001	N/A
Male sex	620 (47.8)	343 (56.5)	276 (40.4)	<0.001	<0.001
Days to sample, median (IQR)	5 (3–12)	5 (3–11)	5 (2–12)	0.69	0.44
Hypertension	766 (59.1)	307 (50.9)	456 (67)	<0.001	0.007
Diabetes mellitus	157 (12.1)	62 (10.3)	94 (13.8)	0.07	0.002
Previous history of stroke	168 (13.1)	66 (10.9)	102 (15)	0.04	0.64
Previous history of MI	128 (10)	31 (5.1)	97 (14.2)	<0.001	<0.001
Previous history of PAD	104 (8.1)	24 (4)	80 (11.7)	<0.001	<0.001
Atrial fibrillation	226 (17.6)	70 (11.6)	156 (22.9)	<0.001	0.03
Current smoker	206 (16)	137 (22.7)	69 (10.1)	<0.001	0.16
Hyperlipidaemia	412 (32.1)	175 (13.6)	237 (18.5)	0.03	0.001
Previous antiplatelet therapy	513 (40)	185 (30.6)	328 (48.3)	<0.001	0.01
Previous antihypertensive therapy	750 (58.5)	278 (46)	472 (69.5)	<0.001	<0.001
Previous statin therapy	312 (24.3)	108 (17.9)	204 (30)	<0.001	<0.001
NIHSS index event, median (IQR)	1 (0–4)	1 (0–3)	1 (0–4)	0.35	<0.001
Cause of stroke				<0.001	0.20
Cardioembolism	258 (20.1)	88 (14.6)	170 (25)		
Large artery	143 (11.1)	57 (9.5)	86 (12.6)		
Small artery	194 (15.1)	107 (17.7)	87 (12.8)		
Undetermined cause	477 (37.1)	243 (40.3)	234 (34.4)		
Unknown	86 (6.7)	35 (5.8)	51 (7.5)		
Multiple	25 (1.9)	8 (1.3)	17 (2.5)		
Other	32 (2.5)	23 (3.8)	9 (1.3)		
PICH	47 (3.7)	27 (4.5)	20 (2.9)		
SAH	22 (1.7)	15 (2.5)	7 (1)		

*Numbers are n (%) unless otherwise stated.

†P values are from χ^2^ tests or Mann-Whitney U tests, as appropriate. Logistic/linear regression was used to adjust for age.

CKD, chronic kidney disease; MI, myocardial infarction; NIHSS, National Institutes of Health Stroke Scale; PAD, peripheral artery disease; PICH, primary intracranial haemorrhage; SAH, subarachnoid haemorrhage; TIA, transient ischaemic attack.

### Cross-correlations between biomarkers

Correlations between biomarker levels are shown in online supplemental table 2. There was good cross-correlation within the subset of inflammatory markers, the greatest was between IL-6 and sTNF-R1 (R=0.47). Within the thrombotic subset, fibrinogen correlated well with vWF (R=0.42) and D-dimer (R=0.41) but none of these factors correlated with P-selectin, TM, PZ or anti-PC antibodies. NSE, a marker of neuronal cell damage, correlated with BDNF (R=0.47). Moderate correlations existed between biomarker subsets, the greatest was between IL-6 and D-dimer (R=0.50), and between sTNF-R1 and D-dimer (R=0.54).

### The relationship between biomarker levels and eGFR

The median (IQR) levels of biomarkers according to eGFR categories are described in table 2. There were significant differences in levels in the inflammatory, thrombotic and cardiac biomarker subsets. IL-6, NGAL, sTNF-R1, TM, fibrinogen, vWF, D-dimer, NT-proBNP and hFABP levels all increased with declining eGFR (all p<0.001).

**Table 2:** Median (IQR) levels of biomarkers according to eGFR category (mL/min/1.73 m^2^)

Biomarker	GFR ≥90	GFR 60–89	GFR 30–59	GFR <30	P value*
	Median (IQR)	Median (IQR)	Median (IQR)	Median (IQR)	
Inflammatory markers					
IL-6, pg/mL	1.8 (0.6–3.6)	1.7 (0.9–4.7)	2.2 (1.1–6.3)	4.9 (2.6–12.6)	<0.001
CRP, mg/L	2.8 (1.3–6.1)	2.1 (1.1–4.1)	2.4 (1.2–4.9)	3.3 (1.3–8.4)	0.04
NGAL, ng/mL	646.7 (389.4–887.3)	631.9 (462.8–837.3)	768.1 (580.4–954.0)	978.6 (856.1–1151.0)	<0.001
sTNF-R1, ng/mL	0.7 (0.6–1.0)	0.8 (0.6–1.0)	1.0 (0.7–1.4)	2.1 (1.9–3.1)	<0.001
Thrombotic or antiatherogenic markers					
TM, ng/mL	1.4 (1.1–1.7)	1.5 (1.3–1.8)	1.8 (1.4–2.2)	2.7 (2.3–4.0)	<0.001
Fibrinogen, g/L	366.0 (323.0–481.3)	384.5 (319.8–468.0)	395.5 (331.0–482.0)	481.5 (394.5–569.8)	<0.001
vWF, IU/mL	145.0 (114.0–217.0)	149.0 (119.0–191.5)	174.5 (137.0–217.3)	233.0 (203.0–268.0)	<0.001
P-selectin, ng/mL	41.0 (25.0–58.5)	32.0 (16.0–46.0)	36.0 (22.0–52.0)	32.0 (17.0–48.0)	0.002
PZ, ng/mL	1.7 (1.4–2.2)	1.6 (1.3–2.1)	1.7 (1.2–2.1)	1.6 (1.1–2.1)	0.64
D-dimer, ng/mL	157.3 (84.6–303.4)	184.5 (111.1–333.2)	272.1 (183.8–446.1)	412.5 (303.3–627.1)	<0.001
Anti-PC, U/mL	42.2 (20.6–61.1)	43.6 (27.4–71.4)	39.4 (24.4–71.4)	38.0 (28.4–61.5)	0.67
ADAMTS-13, U/mL	84.4 (65.6–103.7)	87.2 (68.7–103.4)	85.4 (66.6–98.9)	76.4 (50.1–98.6)	0.13
Markers of cardiac or neuronal function/injury					
NT-proBNP, pmol/L	332.5 (147.0–597.3)	411.0 (196.8–939.0)	820.0 (399.0–1574.5)	2350.0 (1342.0–4354.0)	<0.001
hFABP, ng/mL	1.9 (1.4–2.9)	2.4 (1.8–3.3)	4.2 (2.6–5.4)	8.8 (6.1–12.7)	<0.001
NSE, ng/mL	7.1 (3.7–11.1)	7.2 (4.6–11.4)	6.9 (4.7–11.1)	9.1 (5.4–12.4)	0.20
BDNF, pg/mL	662.9 (359.8–1543.1)	972.2 (547.4–1597.1)	890.8 (512.7–1446.0)	841.9 (565.7–1758.3)	0.18

*Mann-Whitney U test.

Anti-PC, antiphosphorylcholin; BDNF, brain-derived neurotrophic factor; CRP, C-reactive protein; hFABP, heart-type fatty-acid-binding protein; IL-6, interleukin-6; NGAL, neutrophil-gelatinase-associated lipocalin; NSE, neuron-specific enolase; NT-proBNP, N-terminal pro-B-type natriuretic peptide; PZ, Protein Z; sTNF-R1, soluble tumour necrosis factor receptor-1; TM, thrombomodulin; vWF, von Willebrand factor.

**Figure 1:**
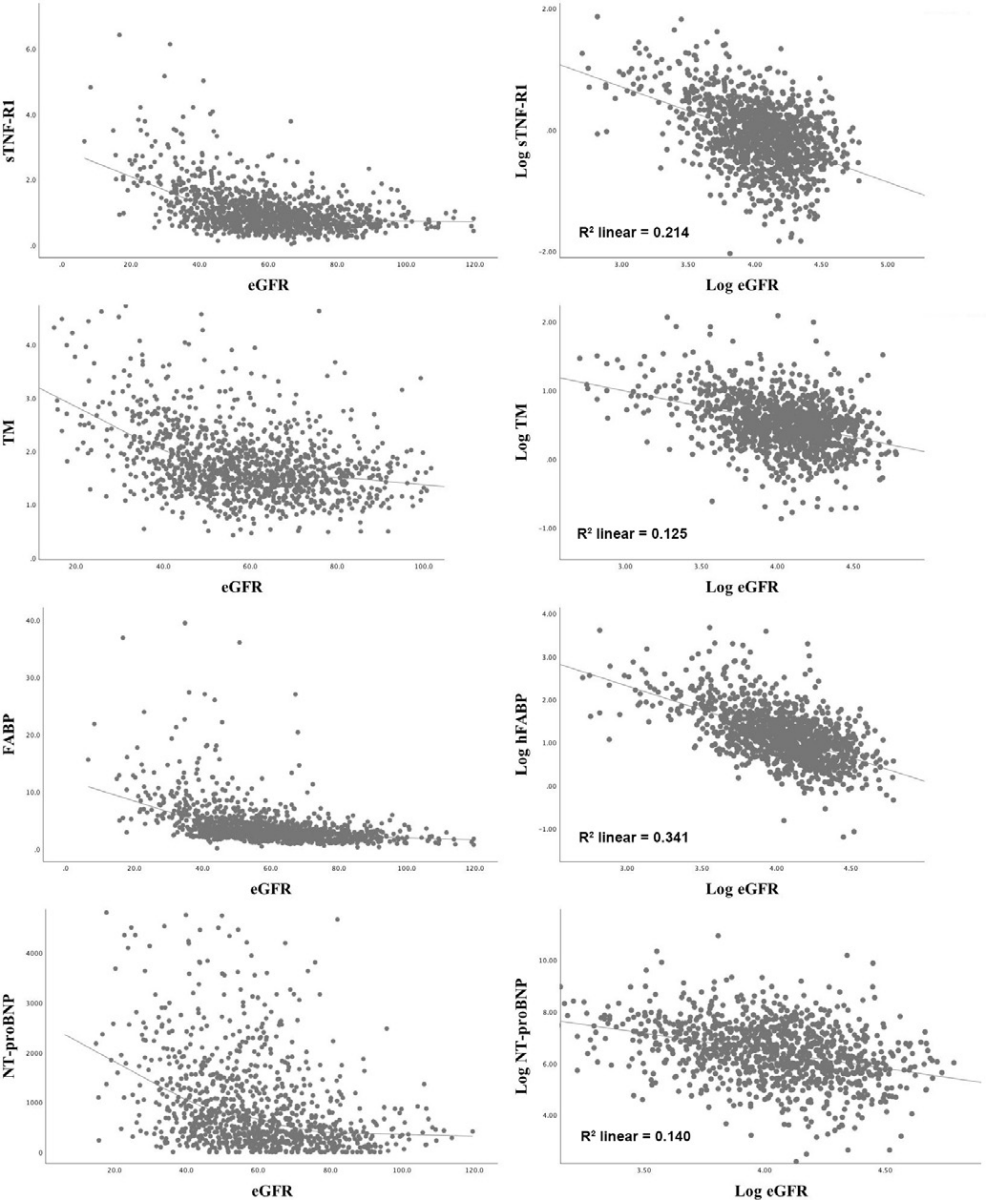
Non-linear and log-linear correlations between sTNF-R1, TM, hFABP, NT-proBNP and eGFR. eGFR, estimated glomerular filtration rate; hFABP, heart-type fatty acid binding protein; NT-proBNP, N-terminal pro-B-type natriuretic peptide; sTNF-R1, soluble tumour necrosis factor receptor-1;TM, thrombomodulin.

The relationship between biomarker levels and eGFR was examined using scatter-plot graphs. Log transformation of both biomarker levels and eGFR highlighted some linear relationships, examples of which are shown in figure 1. Correlations between biomarker levels and eGFR were first assessed using Spearman rank correlation analysis (table 3). There were moderate correlations between the inflammatory markers NGAL and eGFR (R=−0.26), and sTNF-R1 and eGFR (R=−0.41). Within the thrombotic biomarker subset, TM and D-dimer also moderately correlated with eGFR (R=−0.35 and −0.34, respectively). There were also moderate-to-large correlations between markers of cardiac injury, NT-proBNP and hFABP and eGFR (R=−0.38 and −0.56, respectively). In subgroup analysis of the strongest correlations with eGFR, hFABP and sTNF-R1, there were no significant differences according to sex or hypertension status. However, the associations were stronger in older age categories (>65 years, R=−0.50 vs −0.27 and R=−0.37 vs −0.14, for hFABP and sTNF-R1, respectively; p<0.001).

**Table 3:** Correlations of biomarker levels with eGFR on both linear and log–log scales using Spearman rank and Pearson correlations

Biomarker	eGFR		Log eGFR		Log eGFR	
	R (unadjusted)	P value	R^2^ (unadjusted)	P value	R^2^ (age-adjusted)	P value
Inflammatory markers						
IL-6	−0.18	<0.001	0.03	<0.001	0.002	0.18
CRP	−0.07	0.049	0.01	0.02	0.001	0.29
NGAL	−0.26	<0.001	0.06	<0.001	0.03	<0.001
sTNF-R1	−0.41	<0.001	0.21	<0.001	0.12	<0.001
Thrombotic or antiatherogenic markers						
TM	−0.35	<0.001	0.13	<0.001	0.10	<0.001
Fibrinogen	−0.15	<0.001	0.02	<0.001	0.01	0.02
vWF	−0.27	<0.001	0.08	<0.001	0.02	<0.001
P-selectin	−0.08	0.008	0.003	0.05	0.001	0.33
PZ	0.03	0.298	0.001	0.48	0.001	0.35
D-dimer	−0.34	<0.001	0.11	<0.001	0.01	0.01
Anti-PC	0.05	0.125	0.002	0.21	0.003	0.13
ADAMTS-13	0.09	0.008	0.007	0.02	0.0008	0.40
Markers of cardiac or neuronal function/injury						
NT-proBNP	−0.38	<0.001	0.14	<0.001	0.05	<0.001
hFABP	−0.56	<0.001	0.34	<0.001	0.20	<0.001
NSE	−0.08	0.009	0.01	0.003	0.005	0.02
BDNF	0.01	0.852	0	0.99	0.0002	0.6

Anti-PC, antiphosphorylcholin; BDNF, brain-derived neurotrophic factor; CRP, C-reactive protein; hFABP, heart-type fatty-acid-binding protein; IL-6, interleukin-6; NGAL, neutrophil-gelatinase-associated lipocalin; NSE, neuron-specific enolase; NT-proBNP, N-terminal pro-B-type natriuretic peptide; PZ, Protein Z; sTNF-R1, soluble tumour necrosis factor receptor-1; TM, thrombomodulin; vWF, von Willebrand factor.

Correlation coefficients were also compared between TIA, minor and major stroke subgroups in online supplemental table 3. There were no significant differences apart from CRP, fibrinogen and D-dimer, but the strength of these correlations did not increase in the major stroke subgroup. Unadjusted and age-adjusted correlations of log-biomarker levels and log-eGFR were then performed using Pearson correlation analysis (table 3). After adjustment for age, log-eGFR similarly correlated mostly strongly and significantly with log-sTNF-R1 (R^2^=0.12), log-TM (R^2^=0.10), log-NT-proBNP (R^2^=0.05) and log-hFABP (R^2^=0.20, all p<0.001). There were little or no correlations between the other log-biomarkers and log-eGFR after adjustment. When the correlations were repeated using eGFR-FAS, the results were similar in all analyses (online supplemental table 4).

### Biomarker levels, eGFR and all-cause mortality

During 9628 patient-years of follow-up (median 7.7 years; IQR=5.8–9.4), there were 567 deaths (43.7% of the population). CKD was associated with all-cause death (unadjusted HR=1.97, 1.68–2.32; p<0.001), however, this association became non-significant after adjustment for age and sex (adjusted HR=1.04, 0.87–1.23; p=0.69). The associations of biomarkers with all-cause death are shown in table 4. After fully adjusting for demographics (age, sex), previous therapy (antiplatelet agents, antihypertensive agents, statin therapy) and risk factors (hypertension, diabetes mellitus, atrial fibrillation, smoking, previous stroke, previous myocardial infarction, previous peripheral artery disease, hyperlipidaemia), IL-6, sTNF-R1, fibrinogen, vWF, P-Selectin, D-dimer, NT-proBNP, hFABP, NSE and BDNF all remained predictive of death of any cause. However, adjustment for GFR specifically did not attenuate any of the associations with all-cause death.

**Table 4:** Univariate and multivariate associations (according to three models*) of each log-biomarker with risk of all-cause death

Biomarker†	Unadjusted		Model 1	Model 2	Model 3	P value
	HR (95% CI)	P value	HR (95% CI)	HR (95% CI)	HR (95% CI)	
Inflammatory markers						
IL-6	1.34 (1.24 to 1.45)	<0.001	1.33 (1.24 to 1.45)	1.33 (1.23 to 1.44)	1.31 (1.20 to 1.42)	<0.001
CRP	1.09 (0.98 to 1.22)	0.12	1.11 (0.99 to 1.25)	1.11 (0.99 to 1.24)	1.12 (0.99 to 1.25)	0.07
NGAL	1.15 (1.03 to 1.29)	0.01	1.11 (0.99 to 1.24)	1.14 (1.02 to 1.28)	1.10 (0.98 to 1.24)	0.09
sTNF-R1	1.43 (1.29 to 1.57)	<0.001	1.34 (1.21 to 1.48)	1.42 (1.27 to 1.58)	1.38 (1.24 to 1.54)	<0.001
Thrombotic or antiatherogenic markers						
TM	1.07 (0.97 to 1.18)	0.20	1.01 (0.91 to 1.12)	1.03 (0.93 to 1.15)	1.04 (0.93 to 1.16)	0.50
Fibrinogen	1.26 (1.13 to 1.41)	<0.001	1.24 (1.11 to 1.39)	1.24 (1.11 to 1.39)	1.23 (1.10 to 1.37)	<0.001
vWF	1.43 (1.28 to 1.60)	<0.001	1.38 (1.24 to 1.55)	1.40 (1.25 to 1.57)	1.40 (1.25 to 1.57)	<0.001
P-selectin	1.17 (1.06 to 1.28)	0.001	1.18 (1.07 to 1.30)	1.18 (1.07 to 1.30)	1.17 (1.07 to 1.29)	0.001
PZ	0.96 (0.88 to 1.05)	0.34	0.98 (0.89 to 1.07)	0.98 (0.90 to 1.07)	0.98 (0.89 to 1.07)	0.59
D-dimer	1.41 (1.27 to 1.56)	<0.001	1.30 (1.17 to 1.45)	1.31 (1.18 to 1.47)	1.34 (1.20 to 1.50)	<0.001
Anti-PC	0.96 (0.87 to 1.05)	0.35	0.99 (0.90 to 1.09)	1.00 (0.91 to 1.10)	0.98 (0.89 to 1.08)	0.72
ADAMTS-13	0.88 (0.80 to 0.98)	0.02	0.93 (0.84 to 1.03)	0.93 (0.84 to 1.03)	0.93 (0.83 to 1.03)	0.17
Markers of cardiac or neuronal function/injury						
NT-proBNP	1.45 (1.29 to 1.62)	<0.001	1.34 (1.20 to 1.50)	1.37 (1.22 to 1.54)	1.34 (1.18 to 1.52)	<0.001
hFABP	1.49 (1.36 to 1.64)	<0.001	1.40 (1.27 to 1.55)	1.59 (1.42 to 1.79)	1.59 (1.42 to 1.79)	<0.001
NSE	1.17 (1.08 to 1.27)	<0.001	1.19 (1.09 to 1.29)	1.19 (1.10 to 1.30)	1.19 (1.10 to 1.30)	<0.001
BDNF	1.09 (1.01 to 1.17)	0.04	1.12 (1.04 to 1.21)	1.12 (1.03 to 1.21)	1.10 (1.02 to 1.19)	0.02

*Model 1: adjusted for age and sex; model 2 adjusted for variables in model 1 plus eGFR; model 3 adjusted for variables in model 2 plus hypertension, diabetes mellitus, previous myocardial infarction, previous peripheral vascular disease, previous ischaemic stroke, atrial fibrillation, current smoker, hyperlipidaemia, previous therapy with antiplatelet agents, statins or antihypertensive agents.

†Given per SD(Ln).

Anti-PC, antiphosphorylcholin; BDNF, brain-derived neurotrophic factor; CRP, C-reactive protein; hFABP, heart-type fatty-acid-binding protein; IL-6, interleukin-6; NGAL, neutrophil-gelatinase-associated lipocalin; NSE, neuron-specific enolase; NT-proBNP, N-terminal pro-B-type natriuretic peptide; PZ, Protein Z; sTNF-R1, soluble tumour necrosis factor receptor-1; TM, thrombomodulin; vWF, von Willebrand factor.

## Discussion

In this population-based cohort study, we showed that certain biomarkers related to inflammation (NGAL, sTNF-R1), thrombosis (TM) and cardiac injury (NT-proBNP, hFABP) correlated with renal function in patients with TIA or stroke independently of age. However, the strength of these associations, apart from hFABP, were generally weak, suggesting that putative renal vascular factors such as chronic inflammation, oxidative stress or coagulopathy are unlikely to explain the association between CKD and stroke risk. These findings are consistent with the hypothesis that traditional risk factors, particularly blood pressure, remain mechanistically more meaningful. In a systematic review and meta-analysis of low GFR and stroke risk,^25^ this risk association was greatly attenuated by adjustment for long-term blood pressure burden, suggesting that hypertension is an important confounder of this relationship.

In keeping with earlier studies,^26^ many circulating biomarkers (IL-6, NGAL, sTNF-R1, TM, fibrinogen, VvWF, D-dimer, NT-proBNP and hFABP) were elevated in patients with CKD, increasing with worsening renal function. There was a small correlation between sTNF-R1 and eGFR. sTNF-R1 interacts with its membranous counterpart (mTNFR) leading to a proinflammatory stimulus via activation of nuclear factor kappa B or activator protein 1.^27^ It has previously been associated with renal progression, cardiovascular events and all-cause mortality in patients with CKD regardless of the underlying cause.^28 29^

Even after adjustment for age, there was still a moderate correlation between TM and eGFR. TM is a vasculoprotective transmembrane glycoprotein that has both anticoagulant and anti-inflammatory activity.^30^ It can also be released/shed from the endothelium as an extracellular soluble form, indicative of inflammatory cellular damage. In an unadjusted analysis of 59 children with CKD, TM has been previously shown to be strongly correlated with eGFR as well as other markers of endothelial dysfunction and oxidative stress such as asymmetric dimethylarginine and serum oxidised low-density lipoprotein (LDL).^31^ In the studied children with higher TM concentrations, significantly higher albuminuria was also found. Although albuminuria was not measured in this study, it is postulated to be a surrogate biomarker of generalised endothelial dysfunction associated with increased risk of cardiovascular events such as stroke.^32^

Some of the stronger biomarker-eGFR correlations in this study, after adjustment, were those of the cardiac injury markers, NT-proBNP and hFABP, particularly in the case of the latter. NT-proBNP is a peptide secreted from the cardiac ventricles in response to increasing tension in the ventricular wall.^33^ It is also a diagnostic and prognostic tool in congestive heart failure. Since NT-proBNP clearance occurs only in the kidney, elevated levels may result from decreasing renal function because of increased intravascular volume, in addition to impaired cardiac function.^34^ Extravascular volume expansion is known to be an important mechanism in the pathophysiology of hypertension in CKD,^35^ which may explain the correlation in the setting of TIA or stroke in this study. Similarly, hFABP is a marker of myocardial injury and heart failure.^36^ Although it is mostly expressed in the heart and skeletal muscle, hFABP has also been described in human glomeruli,^37^ localised largely along the capillary wall and appears to be associated with proteinuria in obese patients in a process might be related to podocytes and lipid dysmetabolism.^38^

The reported correlations were not confounded by stroke severity as the strength of correlation between these biomarkers and eGFR did not generally differ between TIA, minor or major stroke group. However, given the strong cross-correlation within biomarker subsets, if renal function was truly associated with inflammatory or thrombotic processes at the time of a vascular event, then eGFR should correlate similarly with all biomarkers within a subset instead of with individual ones without a clear pattern of association. Furthermore, many of these circulating biomarkers including NGAL, TM and NT-proBNP are elevated in patients with CKD and appear to rise contemporaneously with a drop in GFR.^39–41^ Thus, their interpretation in this setting may be unclear as elevated levels may not necessarily reflect increased production but rather a prolonged half-life caused by impaired clearance.

Similar to previous systematic review and meta-analysis,^42^ we found that the association of CKD and all-cause mortality was greatly attenuated with adjustment for age and sex. We did not find that eGFR confounded the previously reported associations between certain biomarkers and all-cause mortality, namely sTNF-R1, vWF, hFABP and NT-proBNP.^19^ Our findings are in keeping with earlier work demonstrating that inflammatory biomarkers such as IL-6, TNF-alpha, fibrinogen and albumin are associated with mortality independently of eGFR, even within the CKD population.^18 43^

Our study had a number of limitations. First, we were unable to measure all biomarkers in the entire patient cohort. Second, we may have overestimated the prevalence of CKD in this study since GFR was estimated from baseline creatinine at the time of the vascular event, and acute kidney injury (AKI) is a common complication following stroke.^44^ However, most of the included patients had either a TIA or minor stroke which are not frequently associated with as many systemic sequelae such as AKI. Third, with only one-time measurement of each parameter, some dilution of correlations is possible. Fourth, urine albumin was not measured in this study and we have previously shown that the relationship between albuminuria and stroke may be mechanistically different to that of low eGFR and stroke.^45^

As the global burden of CKD rises,^46^ so too will its potential contribution to stroke mechanisms and its prevalence in stroke survivors. However, disentangling the pathophysiology of stroke in CKD and the importance of non-traditional or renal-specific risk factors has been challenging.^47^ Large studies such as this one with a population-based design help provide further insights into potential causal pathways and the importance of adjustment for confounders such as age. Most older adults develop ‘inflammageing’, a condition characterised by elevated levels of blood inflammatory markers that carries high susceptibility to cardiovascular disease, which is not specific to CKD.^48^

In conclusion, there were some correlations between biomarkers related to inflammation, thrombosis and cardiac injury with renal function in the setting of TIA or stroke. The correlations with cardiac biomarkers may represent volume expansion and higher blood pressure at the time of the vascular event. Endothelial dysfunction in CKD may also play a role since TM and hFABP did correlate with eGFR, and have both previously been associated with albuminuria, a sensitive biomarker of endotheliopathy. However, the strength of the correlations were generally weak or attenuated with adjustment for age, and any putative causal relationships may be confounded by impaired biomarker excretion in advanced kidney disease. Further studies should investigate the association between these biomarkers and albuminuria in the setting of cerebrovascular events.
