# Supplementary material for: Associations of blood biomarkers with glomerular filtration rate in patients with TIA and stroke: population-based study
Source: Stroke Vasc Neurol. 2020 Sep 3;6(1):48–56. doi: 10.1136/svn-2020-000422 (PMC8005904; doi:10.1136/svn-2020-000422)
Supplement: Supplementary data [file svn-2020-000422supp001.pdf]

ONLINE SUPPLEMENTAL MATERIALS

Associations of Blood Biomarkers with Glomerular Filtration Rate in Patients with TIA and Stroke: Population-Based Study

Dearbhla M. Kelly, MBBChBAO MSc MRCPI,[1] Linxin Li, DPhil,[1] Annette I. Burgess, DPhil,[1] Debbie L. Poole, HNC,[1] Julia M. Duerden, PhD[1] Peter M. Rothwell, MD PhD FRCP FMedSci[1] on behalf of the Oxford Vascular Study.

[1]Wolfson Center for Prevention of Stroke and Dementia, Nuffield Department of Clinical Neurosciences, John Radcliffe Hospital, University of Oxford, United Kingdom.

Corresponding author:

Professor Peter Rothwell,

Wolfson Center for Prevention of Stroke and Dementia, Nuffield Department of Clinical Neurosciences, John Radcliffe Hospital, University of Oxford, United Kingdom.

Tel no: +441865231601

Email: peter.rothwell@ndcn.ox.ac.uk

Kelly DM, *et al. Stroke Vasc Neurol* 2021; 6:e000422. doi: 10.1136/svn-2020-000422

TABLE OF CONTENTS

Page Number

Supplementary Tables

Table I: Biomarker Measurement Methods and Intra- and inter-assay coefficients of variation……………………………………………………………3

Table II: Correlations of biomarkers (Spearman rank correlation, p-value)…………...4

Table III: Correlations of biomarker levels with eGFR in TIA, minor and major stroke subgroups (Spearman rank correlation, p-value)………………………………...5

Table IV: Correlations of biomarker levels with eGFR-FAS (eGFR estimated using the Full Age Spectrum) on both linear and log-log scales using Spearman rank and Pearson correlations…………………………………………………………………6

Supplementary Table I: Biomarker Measurement Methods and Intra- and inter-assay coefficients of variation

Biomarker	N	Measurement System	Intra-assay CV%	Inter-assay CV%
Inflammatory markers				
IL-6, pg/ml	1158	Randox microchip	21	13.4
CRP, mg/l	914	Randox microchip	5.7	11.1
NGAL, ng/ml	977	Randox microchip	4.7	9.0
sTNFR-1, ng/ml	1210	Randox microchip	7.8	14.1
Thrombotic or anti-atherogenic Markers				
TM, ng/ml	1213	Randox microchip	10.1	14.1
Fibrinogen, g/L	1036	Stago analyzer	2.3	5.7
vWF, iu/ml	1047	Stago analyzer	4.6	4.6
P-selectin, ng/ml	1059	ELISA	4.9	9.0
PZ, ng/ml	1021	ELISA	4.9	14.8
D-dimer, ng/ml	1189	Stago analyzer	4.3	4.3
Anti-PC, U/ml	901	ELISA	6.7	8.2
ADAMTS-13, U/ml	865	ELISA	8.9	8.2
Markers of cardiac or neuronal function/injury				
Nt-proBNP, pmol/l	1049	ELISA	8.6	10.1
hFABP, ng/ml	1213	Randox microchip	14.6	10.8
NSE, ng/ml	1211	Randox microchip	7.5	20.0
BDNF, pg/ml	1208	Randox microchip	9.6	11.1

Total samples analysed differ because of constraints on sample size, where warfarin may have affected protein synthesis, and according to the materials available. CV indicates coefficient of variation, IL-6, interleukin-6; CRP, C-reactive protein; NGAL, neutrophil gelatinase-associated lipocalin; sTNFR-1, soluble tumor necrosis factor receptor type 1; TM, thrombomodulin; vWF, von Willebrand Factor; PZ, protein Z; anti-PC, anti- phosphorylcholine antibodies; PT, prothrombin time; APTT, activated partial thromboplastin time; Nt-proBNP, N-terminal pro-B-type natriuretic peptide; hFABP, heart type fatty acid binding protein; NSE, neurone specific enolase; BDNF, brain-derived neurotrophic factor.

Supplementary Table II: Correlations of biomarkers (Spearman rank correlation, p-value)

	CRP	NGAL	sTNFR-1	TM	Fibrinogen	vWF	P-selectin	PZ	D-dimer	Anti-PC	ADAMTS-13	NT-proBNP	hFABP	NSE	BDNF
**Il-6**	0.46	0.35	0.47	0.13	0.45	0.46	0.18	-0.18	0.50	-0.08	-0.30	0.29	0.39	0.24	0.23
	<0.001	<0.001	<0.001	<0.001	<0.001	<0.001	<0.001	<0.001	<0.001	0.06	<0.001	<0.001	<0.001	<0.001	<0.001
**CRP**		0.25	0.33	0.08	0.47	0.29	0.11	-0.02	0.33	0.001	-0.09	0.13	0.21	0.11	0.12
		<0.001	<0.004	0.01	<0.001	<0.001	0.01	0.74	<0.001	0.98	0.06	<0.001	<0.001	0.001	<0.001
**NGAL**			0.45	0.25	0.21	0.23	0.19	-0.14	0.36	0.01	-0.14	0.18	0.31	0.30	0.38
			<0.001	<0.001	<0.001	<0.001	<0.001	0.001	<0.001	0.90	0.003	<0.001	<0.001	<0.001	<0.001
**sTNFR-1**				0.42	0.32	0.42	0.19	-0.14	0.54	-0.10	-0.20	0.32	0.53	0.21	0.17
				<0.001	<0.001	<0.001	<0.001	<0.001	<0.001	0.01	<0.001	<0.001	<0.001	<0.001	<0.001
**TM**					0.01	0.15	0.06	-0.02	0.24	-0.08	-0.02	0.10	0.33	0.09	0.02
					0.89	<0.001	0.13	0.61	<0.001	0.05	0.64	0.001	<0.001	0.002	0.56
**Fibrinogen**						0.42	0.09	-0.06	0.41	-0.03	0.002	0.17	0.24	0.11	0.15
						<0.001	0.004	0.07	<0.001	0.33	0.95	<0.001	<0.001	0.003	<0.001
**vWF**							0.12	-0.09	0.47	-0.06	-0.19	0.30	0.43	0.15	0.12
							<0.001	0.01	<0.001	0.07	<0.001	<0.001	<0.001	<0.001	0.001
**P-selectin**								-0.01	0.22	-0.01	-0.01	0.09	0.13	0.14	0.14
								0.73	<0.001	0.73	0.84	0.01	<0.001	<0.001	<0.001
**Protein Z**									-0.11	0.03	0.07	-0.07	-0.09	-0.03	-0.06
									0.004	0.40	0.03	0.40	0.02	0.41	0.14
**D-dimer**										-0.10	-0.30	0.35	0.44	0.20	0.16
										0.02	<0.001	<0.001	<0.001	<0.001	<0.001
**Anti-PC**											0.16	0.03	-0.09	0.07	-0.003
											<0.001	0.36	0.32	0.11	0.94
**ADAMTS-13**												-0.04	-0.23	-0.09	-0.14
												0.24	<0.001	0.03	0.001
**NT-proBNP**													0.31	0.08	-0.04
													<0.001	0.007	0.17
**hFABP**														0.17	0.14
														<0.001	<0.001
**NSE**															0.47
															<0.001

Supplementary Table III: Correlations of biomarker levels with eGFR in TIA, minor and major stroke subgroups (Spearman rank correlation, p-value)

	TIA		Minor Stroke (NIHSS≤ 3)		Major Stroke (NIHSS>3)		
	eGFR		eGFR		eGFR		
**Biomarker**	**R**	**p-value**	**R**	**p-value**	**R**	**p-value**	**P value for difference**
Inflammatory markers							
Il-6	-0.18	0.001	-0.25	<0.001	-0.13	0.395	0.23
CRP	-0.09	0.086	-0.12	0.016	0.13	0.129	0.04
NGAL	-0.28	<0.001	-0.31	<0.001	-0.20	0.005	0.41
sTNFR-1	-0.36	<0.001	-0.46	<0.001	-0.46	<0.001	0.15
Thrombotic or anti-atherogenic markers							
TM	-0.29	<0.001	-0.34	<0.001	-0.43	<0.001	0.11
Fibrinogen	-0.20	<0.001	-0.22	<0.001	-0.03	0.666	0.03
vWF	-0.35	<0.001	-0.26	<0.001	-0.25	<0.001	0.29
P-selectin	-0.10	0.078	-0.08	0.128	-0.09	0.103	0.96
Protein Z	0.11	0.056	-0.04	0.409	0.05	0.352	0.13
D-dimer	-0.43	<0.001	-0.42	<0.001	-0.18	0.003	<0.001
Anti-PC	0.08	0.173	0.09	0.087	-0.06	0.412	0.20
ADAMTS-13	0.17	0.002	0.06	0.301	0.01	0.856	0.16
Markers of cardiac or neuronal function/injury							
NT-proBNP	-0.32	<0.001	-0.42	<0.001	-0.39	<0.001	0.29
hFABP	-0.56	<0.001	-0.60	<0.001	-0.61	<0.001	0.55
NSE	0.01	0.845	-0.14	0.001	-0.05	0.391	0.08
BDNF	0.04	0.459	-0.02	0.726	0.09	0.141	0.54

IL-6, interleukin-6; CRP, C-reactive protein; NGAL, neutrophil gelatinase-associated lipocalin; sTNFR-1, soluble tumor necrosis factor receptor type 1; TM, thrombomodulin; vWF, von Willebrand Factor; PZ, protein Z; anti-PC, anti- phosphorylcholine antibodies; PT, prothrombin time; APTT, activated partial thromboplastin time; Nt-proBNP, N-terminal pro-B-type natriuretic peptide; hFABP, heart type fatty acid binding protein; NSE, neurone specific enolase; BDNF, brain-derived neurotrophic factor; NIHSS, National Institutes of Health Stroke Scale.

Kelly DM, *et al. Stroke Vasc Neurol* 2021; 6:e000422. doi: 10.1136/svn-2020-000422

Supplementary Table IV: Correlations of biomarker levels with eGFR-FAS (eGFR estimated using the Full Age Spectrum) on both linear and log-log scales using Spearman rank and Pearson correlations

	**eGFR**		**Log eGFR**		**Log eGFR**	
**Biomarker**	**R (unadjusted)**	**p-value**	**R^2 (unadjusted)**	**p-value**	**R^2 (age-adjusted)**	**p-value**
Inflammatory markers						
Il-6	-0.23	<0.001	0.05	<0.001	0.001	0.23
CRP	-0.08	0.02	0.008	0.009	0.001	0.29
NGAL	-0.27	<0.001	0.07	<0.001	0.03	<0.001
sTNFR-1	-0.45	<0.001	0.23	<0.001	0.11	<0.001
Thrombotic or anti-atherogenic markers						
TM	-0.35	<0.001	0.11	<0.001	0.09	<0.001
Fibrinogen	-0.18	<0.001	0.02	<0.001	0.01	0.02
vWF	-0.32	<0.001	0.10	<0.001	0.02	<0.001
P-selectin	-0.09	0.005	0.004	0.04	0.001	0.46
Protein Z	0.05	0.08	0.002	0.16	0.001	0.35
D-dimer	-0.43	<0.001	0.17	<0.001	0.006	0.01
Anti-PC	0.09	0.009	0.006	0.02	0.003	0.10
ADAMTS-13	0.14	<0.001	0.01	0.001	0.002	0.25
Markers of cardiac or neuronal function/injury						
NT-proBNP	-0.43	<0.001	0.16	<0.001	0.03	<0.001
hFABP	-0.60	<0.001	0.37	<0.001	0.19	<0.001
NSE	-0.07	0.01	0.007	0.004	0.004	0.03
BDNF	-0.006	0.84	0.00005	0.80	0.0002	0.66

Anti-PC indicates antiphosphorylcholin; BDNF, brain-derived neurotrophic factor; CRP, C-reactive protein; hFABP, heart-type fatty-acid-binding protein; IL6, interleukin-6; NGAL, neutrophil-gelatinase-associated lipocalin; NSE, neuron-specific enolase; NT-proBNP, N-terminal pro-B-type natriuretic peptide; sTNFR-1, soluble tumor necrosis factor α receptor-1; TM, thrombomodulin; and vWF, von Willebrand factor.
